# Support for decision-making guidance in England: a pragmatic review

**DOI:** 10.1093/medlaw/fwaf021

**Published:** 2025-07-31

**Authors:** Jillian Craigie, Antonia Alley, Maria Teresa Cotrufo, Michael Bach, Jodie Rawles, Isabel C H Clare, Matt Matravers, Francesca Happé

**Affiliations:** Centre of Medical Law and Ethics, Dickson Poon School of Law, King’s College London, London, WC2R 2LS, United Kingdom; Carnall Farrar, London, W6 0NB, United Kingdom; Centre of Medical Law and Ethics, Dickson Poon School of Law, King’s College London, London, WC2R 2LS, United Kingdom; School of Disability Studies, Toronto Metropolitan University, Toronto, Canada; Department of Psychiatry, University of Cambridge, Cambridge, CB2 0SZ, United Kingdom; Department of Psychiatry, University of Cambridge, Cambridge, CB2 0SZ, United Kingdom; York Law School, University of York, York, YO10 5GD, United Kingdom; Social, Genetic & Developmental Psychiatry Centre, Institute of Psychiatry, Psychology and Neuroscience, King’s College London, London, SE5 8AF, United Kingdom

## Abstract

Law and policy concerning personal decision-making increasingly recognizes a role for support to enable greater autonomy and legal recognition for adults whose decision-making ability may be limited. Support for decision making (SFDM) is embedded in England and Wales under the Mental Capacity Act 2005 (MCA). It has also gained traction internationally through the UN Convention on the Rights of Persons with Disabilities (CRPD), to which the UK is a signatory. However, these two legal reference points diverge in their understanding of SFDM, which presents challenges for putting it into practice. A pragmatic review methodology identified 40 resources containing SFDM guidance, providing insight into its implementation and conceptualization in England. An analysis indicates the need for authoritative guidance that provides more multifaceted advice, recognizing key variables including: the nature of the decision, source of decision-making difficulties, and the relationship of the supporter. Gaps in guidance provision are also identified for decision-makers, third parties, and the mental health context. The resources largely conceptualize SFDM as a means to enable mental capacity. However, recent developments propose a CRPD-aligned approach that includes SFDM in the context of substituted decisions. This generates a dualistic model of SFDM in England, raising new questions in this area.

## I. INTRODUCTION

The concept of support for decision-making (SFDM) is gaining prominence internationally in public policy.^1^ The broad motivation is the idea that, for adults, the freedom to make our own decisions is crucial to personal autonomy and to the realization of certain human rights, and that in the context of some disabilities and medical conditions, support practices can increase opportunities for being the decision-maker. Those who may be supported in decision-making include people with dementia, brain injury, mental ill-health, or neurodevelopmental disabilities such as intellectual disability and autism. In England and Wales, relevant legal provisions are found in the Mental Capacity Act 2005 (MCA) and the Care Act 2014.^2^ However, insight into the practice of SFDM in this jurisdiction is currently limited.^3^

The present review aims to provide insight into SFDM practice in England, through an analysis of current guidance. Guidance documents are considered a useful platform for the implementation of law, policy, and evidence in practice.^4^ They are a means for promoting consistency and efficiency in care and a method to close the gap between evidence and practice.^5^ Medical professionals have a legal duty to stay up-to-date with developments in practice, including relevant guidance,^6^ and commentators have noted an increasingly important role for such documents in determining the standard of care in cases of alleged clinical negligence in England.^7^ Resources that include guidance on SFDM therefore offer a window into its implementation. The review is pragmatic rather than systematic due to the nature of the documents, which are largely unpublished grey literature.^8^ The findings address a gap in understanding about the kinds of organizations that are producing relevant guidance in England; who it is for; its scope in terms of the people being supported (hereafter decision-makers) and the kinds of decisions concerned; the practical advice that is provided; and its legal grounding.

Insight concerning the legal grounding of guidance is important because of apparent tensions in the conceptualization of SFDM. For the purpose of this review, SFDM is defined as any activity where someone is assisted in decision-making, in the context of any disability or condition. This definition encompasses two legal perspectives that envisage SFDM in importantly different ways. One understands support primarily as a means to enable, and potentially augment, the person’s decision-making abilities. In England and Wales, this perspective is linked to the MCA, which provides that an adult must not be deemed unable to make a decision unless ‘all practicable steps’ have been taken to enable the person’s mental capacity to make the decision, as defined by the Act, ‘without success’.^9^ This requirement is designed to ensure that any interference with Article 8 protections of autonomy under the European Convention of Human Rights, in applications of the MCA, is proportionate.^10^ It is often, though not uncontroversially, described using the term ‘supported decision-making’.^11^

A second perspective on SFDM emphasizes enabling the expression of the person’s will and preferences and is linked to the UN Convention on the Rights of Persons with Disabilities (CRPD). The CRPD mandates that its signatories, which include the UK, ‘take appropriate measures to provide access by persons with disabilities to the support they may require in exercising their legal capacity’,^12^ giving ‘primacy to a person’s will and preferences’.^13^ While not legally bound by the Convention, as a signatory, the UK has expressed an intention to comply with it. The CRPD is playing a significant role internationally in law reform around personal decision-making, with countries including Scotland and Australia having undertaken major reviews that include this area, to move towards CRPD compliance.^14^

Discussions of SFDM in connection with the Convention place much less emphasis on mental capacity, in line with scepticism about the concept expressed by the UN treaty body, the Committee on the Rights of Persons with Disabilities.^15^ However, despite a literature discussing key concepts such as will and preferences, there remains uncertainty about how a CRPD perspective on SFDM translates into practice.^16^ Grappling with the implementation of Article 12, the recent Scottish review of mental health law observed, ‘We believe that there is considerable force in the Committee’s arguments, but there are also some practical difficulties. There is no agreed methodology for assessing “will and preferences” or resolving dilemmas where these are in tension’.^17^ In light of these tensions and uncertainties, the present review sought to examine the legal foundations of current SFDM guidance in England.

An exploration of guidance also provides an opportunity to identify potential gaps or weaknesses in the implementation of SFDM in England. Post-legislative scrutiny of the MCA found that the decision-making support it requires was ‘rare in practice’.^18^ Research into the MCA has suggested that more guidance is needed to help social care practitioners implement its principles, including its support requirement.^19^ The literature advocating for SFDM in connection with the CRPD also acknowledges the benefits of written resources,^20^ with Browning observing that poor practice in SFDM, ‘is often the result of a lack of information and appropriate guidance’.^21^

## II. TWO PERSPECTIVES ON SUPPORT FOR DECISION-MAKING

To ground the analysis and further motivate the work, this section provides a brief account of the identified legal tensions concerning the meaning of SFDM. We propose that these tensions can be understood in terms of a divergence in assumptions about what is essential to adult human agency. Viewed in this way, the different approaches to SFDM are explained at least in part by a philosophical difference that sits in the background of the legal debates.

Linked to the MCA is a perspective that sees certain functional abilities (or decision-making skills)^22^ as essential to adult human agency, and therefore also potentially relevant to legal standing or legal agency (jointly referred to as legal capacity). This perspective connects with some prominent thinking in the philosophical literature. Discussions in this context sometimes concern the psychological elements necessary for personal autonomy, and theorists have pointed to capacities for rational deliberation,^23^ the ability to reflect on and endorse one’s own desires,^24^ and to reflect on one’s life as a whole.^25^

However, some developments in philosophy and cognitive science have challenged these ideas. For example, Jaworska rejected the importance placed by Dworkin on the ability to have a life plan, in the realization of autonomy.^26^ Instead, Jaworska focuses attention on the capacity to value. More recently, Doris has used scientific developments to reject the standard idea that the exercise of agency requires the exercise of reflective agency, understood as ‘*judgement and behaviour ordered by self-conscious reflection about what to think and do*’.^27^ In its place, Doris develops a theory in which the exercise of agency is about the expression of the person’s values, where this ‘need not be a reflective process’.^28^

In different ways, these challenges to orthodox understandings of autonomy and agency resonate with a perspective that is implicit in the CRPD approach to SFDM. On this view, adult human agency is grounded in will and preferences, which is what makes only these features essential for legal standing and legal agency.^29^ The decision-making ‘skills’^30^ or abilities that are considered a core part of adult human agency from the perspective linked to the MCA, may therefore be provided by others as support measures without affecting the person’s legal capacity.^31^ Understood in this way, the finding that someone lacks certain functional decision-making abilities indicates the need for support *rather than* the need to make a decision on their behalf.^32^ SFDM is seen as the answer in place of substituted decision-making, setting up a binary between these two practices.

However, recent developments in the implementation of Article 12 have challenged this way of understanding SFDM, as distinct from substituted decision-making. In a discussion of the issue as part of an Australian Royal Commission report, Bigby and colleagues argue for a conception of SFDM grounded in the CRPD, where ‘support and substitution are not viewed in opposition, but rather as existing along a spectrum’.^33^ This position is motivated by a perspective among respondents in their consultation process, ‘that interpreting the preferences of a person with severe cognitive impairment did not naturally fit within the binary understanding of supported decision-making, suggesting that alternative approaches are necessary’.^34^ Respondents indicated the need to recognize an in-between space that exists in practice, which does not neatly fit the binary understanding of either supported or substituted decision-making.^35^ The resolution proposed by Bigby and colleagues holds that:

support and substitution are on a spectrum where some—but not all—substitute decisions are considered a form of supported decision-making, rather than being in opposition to it. In practice, the relevant marker of supported decision-making is that an individual’s stated or perceived ‘will and preferences’ remain at the centre of the decision.^36^

This sketch of the theoretical landscape provides a backdrop for the findings of this review. In this article, the term SFDM is used instead of supported decision-making, on the basis that SFDM is a broader concept that can be straightforwardly recognized within both the MCA and CRPD.^37^ As defined by the Committee on the Rights of Persons with Disabilities, supported decision-making refers narrowly to legal *regimes* that are contrasted with substituted decision-making regimes.^38^ On this understanding, supported decision-making is not possible under the MCA. However, SFDM can be recognized as support *measures* within the CRPD,^39^ which may be implemented in the context of regulation that allows for substituted decision-making, such as the MCA. The guidance identified in this review uses a variety of terminology to describe SFDM.

## III. METHODOLOGY

A pragmatic review methodology was used due to the nature of the documents, which are largely grey literature. Grey literature tends to be unpublished, web-based, and vast, making it difficult to search systematically using established databases.^40^ Following the approach developed by others, systematic review search methods were adapted to accommodate grey literature.^41^

### A. Eligibility criteria

The review identified materials that included guidance on supporting decisions for any adult with a disability or condition that may affect their decision-making, using the eligibility criteria in Table 1. Among the exclusion criteria was an exclusive focus on shared decision-making. Shared decision-making emphasizes a partnership, generally between a patient and healthcare professional, where a decision is made together, in contrast to SFDM, where the decision-maker is distinguished from the person providing support.^42^

**Table 1.** Eligibility criteria.

Inclusion criteria	Exclusion criteria
Includes advice on SFDM for any person with a disability or condition that may affect their decision-making.	Exclusive focus on shared decision-making.
Published by an organization in England, post-2007.	Published outside of England or pre-2007.
Support aimed at adults (16 years old and over).	Support aimed only at children (under 16 years old).
SFDM advice is either the primary focus or a substantial feature of the document.	SFDM advice is limited, for example, outlining only the principles of the MCA 2005.
Intended for people involved in the provision or practice of SFDM, including decision-makers, support providers, or third parties.	Intended for an academic audience only.
In the case of multiple versions: most recent	In the case of multiple versions: not the most recent.

### B. Search strategy

Resources were collected between January 2020 and June 2024. Initial search and screening activity was carried out between January and February 2020, and was followed up in June 2022, June 2023, and June 2024, to seek new or updated guidance. Final checks took place in May 2025.

The initial search involved two strategies to maximize the range of sources for guidance. A targeted search identified organizations that were considered likely to have or recommend guidance on SFDM. These comprised organizations supporting people with conditions that might affect their decision-making in (*n* = 41), advocacy organizations (*n* = 16), government and local authorities (*n* = 7), organizations providing support for carers (*n* = 5), Royal Colleges (*n* = 5), care providers (*n* = 5), public bodies (*n* = 5), statutory bodies (*n* = 3), and others (*n* = 6). The organizations were identified based on the authors’ existing knowledge and Google searches using key terms. Local authorities were randomly selected from regions across England. Websites for the organizations were searched for relevant content, for example, in the form of web pages or documents. Where guidance could not be found, the organization was contacted via email and/or telephone. Informal conversations were conducted in which organizations were asked about what guidance the organization uses internally or recommends, concerning SFDM. Identified resources were checked for references to other potential materials.

The second strategy involved customized Google searches for resources published on the internet using various combinations of key search terms, for example ‘supported decision-making guidance’ or ‘decision-making support guidance’.^43^ This strategy identified relevant guidance produced by organizations that were not included in the first search strategy. This approach returned large numbers of potentially relevant resources, for example, searching ‘supported decision-making guidance England’ returns over 12,000 results. This strategy therefore involved some reliance on Google’s relevancy ranking.^44^ The sample was judged adequate when the search strategy reached saturation, no longer returning new, relevant materials. Relevant resources were checked for references to other potential materials.

The follow-up search and screening activity again involved two strategies. A targeted strategy returned to the organizations that published resources identified in the initial search, seeking updated or new guidance. The second strategy repeated the customized Google searches for new resources or any that were not previously identified.

### C. Analysis

Identified resources were read to make an initial assessment of the content, and the sample was refined using the eligibility criteria (Table 1).^45^ The resulting guidance was analysed to identify: (i) intended audience; (ii) identified features of the decision-maker (the person being supported); (iii) type(s) of decisions being supported; (iv) recommendations for how to practice SFDM; and (v) references to domestic or international law or related documents, for example, codes of practice or general comments.

## IV. RESULTS

The combined search strategy and eligibility test yielded 40 resources (Table 2) containing relevant guidance. Due to the pragmatic methodology, these resources provide an indicative rather than an exhaustive picture of relevant guidance in England.

**Table 2.** Identified resources containing SFDM guidance.

Resource	Published by	Date	Written for	Law	Decisions	Decision-maker
A carer’s guide to the Mental Capacity Act^a^	Sitra, The Carers Trust and Care Charts UK	2015	Carers	MCA	All	People with dementia
A practical guide to supported decision-making^b^	Paradigm	2023	Family, decision-maker, professionals, friends	MCA, Care Act 2014, CRPD	All	All but particularly people with learning disabilities
Capacity to consent to sexual relations^c^	The British Psychological Society	2025	Practitioner psychologists	MCA, Care Act 2014, Sexual Offences Act 2003	Sexual relations	Not specified
Conducting research with people not having the capacity to consent to their participation: A practical guide for researchers^d^	The British Psychological Society	2020	Researchers	MCA	Participation in research	Not specified
Decision-making and consent^e^	General Medical Council	2020	Medical practitioners	Mental capacity law across UK jurisdictions	Medical	Not specified
Decision-making and mental capacity^f^	National Institute for Health and Care Excellence	2018	Health and social care practitioners, independent advocates, third-party practitioners, service users, families, friends, carers.	MCA	All	Not specified
Did you know? The benefits of supported decision-making (consent)^g^	NHS Resolution	2018	NHS staff	MCA	Medical	Not specified
East Sussex Mental Capacity Multi-Agency Policy and Procedures, Edition 1^h^	East Sussex Safeguarding Adults Board	2019	Health and social care staff	MCA	All	Not specified
Facts for families: Supporting people with profound and multiple learning disabilities^i^	Dimensions	2018	Families and friends	MCA	All	People with profound and multiple learning disabilities
Guidance on money management for people who may lack capacity to make some decisions about how their money is used^j^	ARC (Association for Real Change), Social Care Institute for Excellence	2011	Support staff and managers	MCA	Financial	Not specified
Hft operational standards: Making decisions and consent to support (Appendix 1: Practical Tips on Supporting People to Make their Own Decisions)^k^	Hft	2017	Hft Staff	MCA	All	People with learning disabilities
Good and promising practice guide: Inclusive training about Article 12^l^	IDEA 12, Inclusive Direction of Education for Adults on Article 12	2017	Trainers developing sessions on supported decision-making	CRPD (and international models)	All	Not specified
Independence, choice and risk: a guide to best practice in supported decision-making^m^	Department of Health	2007	Everyone involved in supporting adults (18 +) using health and social care in any setting	MCA	All	Not specified
In the driving seat: A workbook to help me plan my support^n^	In Control	2007	Decision-makers who may need support	None	All	Not specified
Involve me: Practical guide^o^	Mencap	2011	Families, frontline staff, service managers, people who plan and commission services, advocates—including peer advocates, and decision-makers at the national, regional, and local level.	MCA	All	People with profound and multiple learning disabilities
Involving people in their own health and care: Statutory guidance for clinical commissioning groups and NHS England^p^	NHS England	2017	Clinical Commissioning Groups and NHS	MCA	Medical	Not specified
Making banking easier—A guide written by Dosh on how to work with banks to get what you need^q^	Dosh Financial Advocacy	2014	Decision-makers who may need support	MCA, Equality Act 2010	Financial	People with learning disabilities
Making decisions and managing difficult situations^r^	Alzheimer’s Society	2016	Carers	MCA	All	People with dementia
Making decisions: A guide for people who work in health and social care^s^	The Mental Capacity Implementation Programme	2024	Health and social care staff	MCA	All	Not specified
Making everyday, financial, health, and welfare decisions post 16^t^	Down’s Syndrome Association	2021	Families and carers	MCA	All	People with Down’s Syndrome
Making financial decisions: Guidance for assessing, supporting and empowering specific decision-making^u^	Empowerment Matters	2014	Providers of relevant support and banking sector staff	MCA	Financial	Not specified
MCA Principle 2—supported decision-making *MV^v^	Social Care Institute for Excellence	2018	Providers of relevant support	MCA	All	Not specified
Mental Capacity Act 2005 in practice: Learning materials for adult social workers^w^	Department of Health	2015	Social workers	MCA	All	Not specified
Mental Capacity Act 2005: An easy read guide^x^	Local Government Association and NHS England	2015	Decision-makers who may need support	MCA	All	Not specified
Mental Capacity Act code of practice^y^	Department for Constitutional Affairs	2007	Providers of relevant support	MCA	All	Not specified
Mental Capacity Act resource and practice toolkit: Supporting people to make their own decisions^z^	Tri.x	2024	Practitioners working in statutory social care or healthcare	MCA	All	Not specified
Mental Capacity Act practice guidance^aa^	Cumbria County Council	2023	Practitioners in adult social care	MCA	All	Not specified
Mental Capacity Act resource pack^bb^	Mencap	2016	Family	MCA	All	People with learning disabilities
Ethics Toolkit, Mental Capacity Act—England and Wales^cc^	British Medical Association	2024	Medical doctors	MCA, CRPD	All	Not specified
Mental capacity—support decision-making after brain injury^dd^	Headway	2016	Providers of relevant support and third parties	MCA	All	People with brain injury
Mental capacity: supporting people with decisions^ee^	Choice Support	2019	Frontline staff and managers	MCA	All	Not specified
National Mental Capacity Act competency framework^ff^	The National Centre for Post-Qualifying Social Work and Professional Practice	2017	Health and social care Staff	MCA	All	Not specified
PfA Factsheet: The Mental Capacity Act 2005 and supported decision-making^gg^	Preparing for Adulthood (National Development Team for Inclusion, The Council for Disabled Children)	2014	Family, carers and local authorities	MCA, Care Act 2014, Children and Families Act 2014	All	Any young person at risk of exclusion due to disability
Providing Independent Advocacy under the Care Act^hh^	Department of Health	2014	Independent advocates	CRPD, MCA, Care Act 2014, Children and Families Act 2014	All	Not specified
Shropshire, Telford, and Wreklin Multi-Agency Mental Capacity Act Guidance^ii^	NHS Shropshire, Telford and Wreklin Integrated Care Board	2024	Staff and others working in Shropshire and Telford and Wrekin	MCA	All	Not specified
Supported Decision-Making Toolkit for People with Communication Difficulties^jj^	National Mental Capacity Forum, Essex Autonomy Project	2023	Providers of relevant support	MCA	All	People with communication difficulties
Supported Loving Toolkit: Contraception^kk^	Choice Support	2019	Anyone providing support	MCA	Contraception and sexual health	People with learning disabilities and autistic people
Supporting people to make decisions: A guide to the Mental Capacity Act for family carers^ll^	Dimensions	2020	Families with a relative who has learning disabilities and/or experiences autism	MCA	All	People with learning disabilities and/or who experience autism
Thinking ahead: a planning guide for families^mm^	Foundation for People with Learning Disabilities	2013, updated 2015	Families	MCA, Care Act 2014	All (planning for the future)	People with learning disabilities
What makes a good assessment of capacity?^nn^	British Psychological Society	2019	Psychologists	MCA, Care Act 2014	All	Not specified

a N Keir and others, ‘A Carer’s Guide to the Mental Capacity Act’ (Sitra, The Carers Trust and Care Charts UK, 2015) < https://www.newham.gov.uk/downloads/file/1871/a-guide-to-the-mental-capacity-act-for-people-caring-for-someone-with-dementia2016> accessed 26 May 2025.

b Warren and Giles (n 48).

c This guidance was updated in January 2025. C Herbet and others, British Psychological Society ‘Capacity to Consent in Sexual Relations’ (British Psychological Society, 2025) <https://explore.bps.org.uk/content/report-guideline/bpsrep.2025.rep126a> accessed 22 May 2025.

d C Dobson and J Hamilton, ‘Conducting Research with People not Having the Capacity to Consent to their Participation: A Practical Guide for Researchers’ (British Psychological Society, 2020) <https://explore.bps.org.uk/content/report-guideline/bpsrep.2020.rep135> accessed 22 May 2025.

e General Medical Council, ‘Decision-making and Consent’ (updated December 2024, General Medical Council, 2020) <https://www.gmc-uk.org/professional-standards/the-professional-standards/decision-making-and-consent> accessed 22 May 2025.

f National Institute for Health and Care Excellence (n 11).

g NHS Resolution ‘Did you know? The benefits of supported decision making (consent)’ (NHS Resolution, 2018) <https://resolution.nhs.uk/wp-content/uploads/2018/09/Did-you-know-The-benefits-of-supported-decision-making-consent-WEB.pdf> accessed 22 May 2025.

h This document was updated in December 2024, but the SFDM advice remained the same. East Sussex Safeguarding Adults Board, ‘East Sussex Mental Capacity Multi-Agency Policy and Procedures’ (Edition 2, East Sussex Safeguarding Adults Board, 2024) <https://www.eastsussexsab.org.uk/guidance/protocol#Mental%20capacity> accessed 26 May 2025.

i Dimensions, ‘Facts for Families: Supporting people with Profound and Multiple learning disabilities’ (*Dimensions* 2018) <https://dimensions-uk.org/wp-content/uploads/Family-factsheet-Supporting-people-with-profoundmultiple-learning-disabilities.pdf> accessed 22 May 2025.

j J Livingstone, ‘Guidance on Money Management for People Who May Lack Capacity to Make Some Decisions about How Their Money is Used’ (ARC 2011) <https://arcengland.org.uk/wp-content/uploads/2021/11/Guidance-on-Money-Management-for-people-who-may-lack-capacity-.pdf> accessed 23 May 2025.

k Hft, ‘Making Decisions and Consent to Support, Appendix 1: Practical Tips on Supporting People to Make their Own Decisions’ (OS/03/17, Hft 2017)

<https://www.hft.org.uk/wp-content/uploads/2017/08/Making-Decisions-and-Consent-to-Support-Appendix-1-Practical-Tips-on-Supporting-People-to-Make-their-own-Decisions.pdf> accessed 23 May 2025.

l IDEA 12 (n 69).

m Department of Health, ‘Independence, Choice and Risk: A Guide to Best Practice in Supported Decision-making’ (Department of Health, 2007) <https://lx.iriss.org.uk/sites/default/files/resources/Independence%2C%20choice.pdf> accessed 22 May 2025.

n Sanderson and McStravick (n 88).

o Mencap, ‘Involve Me: Practical Guide’ (Mencap, 2011) <https://www.mencap.org.uk/sites/default/files/2016-07/Involve%20Me%20practical%20guide_full%20version.pdf> accessed 22 May 2025.

p NHS England, ‘Involving People in their own Health and Care: Statutory Guidance for Clinical Commissioning Groups and NHS England’ (NHS England 2017) <https://www.england.nhs.uk/publication/involving-people-in-their-own-health-and-care-statutory-guidance-for-clinical-commissioning-groups-and-nhs-england/> accessed 22 May 2025.

q Beckford (n 89).

r Alzheimer’s Society, ‘Making Decisions and Managing Difficult Situations’ (Alzheimer’s Society, 2016) <https://www.alzheimers.org.uk/sites/default/files/2018-10/484LP%20Making%20decisions%20and%20managing%20diff%20situations.pdf> accessed 22 May 2025.

s Office of the Public Guardian, ‘Making-Decisions: A Guide for People Who Work in Health and Social Care’ (The Mental Capacity Implementation Programme, 2024) <https://www.gov.uk/government/publications/health-and-social-care-workers-mental-capacity-act-decisions/making-decisions-about-your-health-welfare-or-finances> accessed 22 May 2025.

t Down’s Syndrome Association, ‘Making Everyday, Financial, Health and Welfare Decisions Post 16’ (Down’s Syndrome Association 2021) <https://www.downs-syndrome.org.uk/wp-content/uploads/2021/04/Making-Decisions-post16.pdf> accessed 22 May 2025.

u J Cowley and S Lee, ‘Making Financial Decisions: Guidance for Assessing, Supporting and Empowering Specific Decision Making’ (Empowerment Matters, 2014) <https://empowermentmatters.co.uk/wp-content/uploads/2014/09/assessing-capacity-financial-decisions-guidance-final.pdf> accessed 22 May 2025.

v Social Care Institute for Excellence, ‘MCA Principle 2 - Supported Decision Making *MV’ (Social Care Institute for Excellence 2018) <https://www.youtube.com/watch?v=HPcr6ux3uGk> accessed 22 May 2025.

w D Bogg and S Chamberlian, ‘Mental Capacity Act 2005 in Practice: Learning Materials for Adult Social Workers’ (Department of Health, 2015) <https://assets.publishing.service.gov.uk/media/5a805412e5274a2e8ab4f83f/Pt1_Mental_Capacity_Act_in_Practice_Accessible.pdf> accessed 22 May 2025.

x C Tarling, ‘Mental Capacity Act 2005: An Easy Read Guide’ (Local Government Association and NHS England, 2015) <https://www.local.gov.uk/sites/default/files/documents/easy-read-guide-pdf-16-pa-2cc.pdf> accessed 22 May 2025.

y Department for Constitutional Affairs (n 46).

z ‘Mental Capacity Act Resource and Practice Toolkit: Supporting People to Make Their Own Decisions’ (Tri.x 2024) < https://mca-adults.trixonline.co.uk/chapter/supporting-people-to-make-their-own-decisions> accessed 22 May 2025.

aa N Smith, C Rankin and J Baldwin, ‘Practice Guidance: Mental Capacity Act 2005’ (Cumbria County Council, 2023) <https://proceduresonline.com/trixcms2/media/18521/practice-guidance-mental-capacity-act.pdf> accessed 23 May 2025. The relevant guidance in this resource is derived from an earlier guide published by Research in Practice. This earlier document includes the CRPD in its reference list but does not mention it in the text of the guide. D Baker and M Malone-Lee, ‘Mental Capacity Act 2005 Decision-making—Care, Support, and Treatment’ (Research in Practice 2021) <https://adultsdp.researchinpractice.org.uk/media/ihhlyz4s/adults_pt_mca_web.pdf> accessed 23 May 2025.

bb Mencap, ‘Mental Capacity Act Resource Pack’ (Mencap 2016) < https://www.mencap.org.uk/sites/default/files/2016-06/mental%20capacity%20act%20resource%20pack_1.pdf> accessed 23 May 2025.

cc British Medical Association (n 70).

dd Headway, ‘Mental Capacity: Supporting Decision-making after Brain Injury’ (Headway 2016)

<https://www.headway.org.uk/media/12041/mental-capacity-supporting-decision-making-after-brain-injury-publication.pdf> accessed 22 May 2025.

ee Choice Support, ‘Mental Capacity: Supporting People with Decisions’ (Choice Support 2017).

ff K Brown, D Bogg and M Lyne, ‘National Mental Capacity Act Competency Framework’ (The National Centre for Post-Qualifying Social Work and Professional Practice 2017) <https://eprints.bournemouth.ac.uk/33832/1/MCA-for-web-2018.pdf> accessed 22 May 2025.

gg Preparing for Adulthood, ‘PfA Factsheet: The Mental Capacity Act 2005 and Supported Decision Making’ (Preparing for Adulthood 2014) <https://councilfordisabledchildren.org.uk/sites/default/files/uploads/files/pfa_factsheet_-_mca.pdf> accessed 22 May 2025.

hh Department of Health (n 68).

ii NHS Shropshire, Telford and Wreklin Integrated Care Board, ‘Shropshire, Telford, and Wreklin Multi-Agency Mental Capacity Act Guidance’ (NHS Shropshire, Telford and Wreklin Integrated Care Board 2024) <https://www.shropshiretelfordandwrekin.nhs.uk/policies/shropshire-multi-agency-mental-capacity-act-guidance-2/> accessed 23 May 2025.

jj H Atkinson, M Jayes and A Volkmer, ‘Supported Decision Making Toolkit for People with Communication Difficulties’ (National Mental Capacity Forum 2023) <https://www.careengland.org.uk/supported-decision-making-toolkit-for-people-with-communication-difficulties/> accessed 23 May 2025.

kk Choice Support, ‘Supported Loving Toolkit: Contraception’ (Choice Support 2019) <https://choicesupport.org.uk/about-us/what-we-do/supported-loving/supported-loving-toolkit/contraception> accessed 23 May 2025.

ll Dimensions, ‘Supporting People to Make Decisions: A Guide to the Mental Capacity Act for Family Carers’ (Dimensions 2020) <https://dimensions-uk.org/wp-content/uploads/Facts-for-Families-Mental-Capacity-Act.pdf> accessed 23 May 2025.

mm C Towers, ‘Thinking Ahead: A Planning Guide for Families’ (Foundation for People with Learning Disabilities 2013, updated 2015) <https://www.togethermatters.org.uk/resources-and-information/#work> accessed 23 May 2025.

nn Herbert and others (n 104).

Three pieces of guidance were commonly pointed to when target organizations were consulted about the guidance they use or recommend: the MCA Code of Practice (MCA Code),^46^ the National Institute for Health and Care Excellence (NICE) guideline on decision-making and mental capacity,^47^ and a guide produced by Paradigm.^48^ The first two of these documents supplement the MCA. All organizations advising or representing practitioners cited these documents as the primary guidance in relation to SFDM. Paradigm is a consultancy, training, and development organization promoting person-centred approaches, particularly for people with a learning disability or autism.

The Paradigm guide was updated in 2023 and only this version was included in the analysis.^49^ A revised draft of the MCA Code was released in 2022,^50^ though the analysis includes only the MCA Code,^51^ as the draft has no legal status. The draft MCA Code is commented on in the discussion.

### A. Intended audience

Most of the resources (36 of 40; 90 per cent) were aimed at people providing support, including those involved in relevant training, planning, policy, commissioning, or the practice of support. Four resources (10 per cent) were aimed at decision-makers (people being supported). Of these, three were aimed at any adult who may need support in decision-making, and one was for adults with a learning disability. Three resources (7.5 per cent) were for third-party professionals who may be involved in enacting supported decisions (eg, those working in banks, healthcare, or the criminal justice system). In most cases, the guidance was intended for just one of these audiences, but a small number of documents were for a combination, for example, anyone providing support *and* banking sector staff.

Among the guidance aimed at those providing support, six resources (15 per cent), including the MCA Code,^52^ NICE,^53^ and Paradigm^54^ documents, did not specify the role of the support provider. However, the majority of resources for those providing support were aimed at people in specified roles, which included: healthcare professionals (10; 25 per cent), social care practitioners (10; 25 per cent), family or friends (9; 22.5 per cent), managers or commissioners (6; 15 per cent), carers (5; 12.5 per cent), advocates (3; 7.5 per cent), psychologists (2; 5 per cent), social workers (1; 2.5 per cent), those providing training for supporters (1; 2.5 per cent), and researchers (1; 2.5 per cent). Many of these resources were for support providers across a number of roles, for example, for healthcare and social care practitioners.

### B. Decision-maker (person being supported)

The majority of resources (27; 67.5 per cent) did not specify a particular disability or medical condition in connection with the decision-maker’s need for SFDM. This was the approach taken in the MCA Code,^55^ NICE,^56^ and Paradigm^57^ documents, though Paradigm as an organization has a focus on people with learning disabilities or autism.

Thirteen resources were about SFDM for a more circumscribed group of decision-makers: people with learning disabilities (6, 15 per cent), profound and multiple learning disabilities (2; 5 per cent), autistic people (2; 5 per cent), people with dementia (2; 5 per cent), Down’s syndrome (1; 2.5 per cent), brain injury (1; 2.5 per cent), and communication difficulties (1; 2.5 per cent). Two documents concerned support relevant for more than one specified disability, combining learning disabilities and autism.

### C. Types of decisions

The majority of resources (30; 75 per cent), including the MCA Code,^58^ NICE,^59^ and Paradigm^60^ guidance, took a generic approach in terms of the nature of the decisions being supported.

A quarter of the resources concerned support for a specified type of decision, which included financial decisions (3; 7.5 per cent), medical decisions (3; 7.5 per cent), sexual relations (1; 2.5 per cent), contraception and sexual health (1; 2.5 per cent), participation in research (1; 2.5 per cent), and planning for the future (1; 2.5 per cent).

### D. Recommendations for the practice of SFDM

The findings about advice for SFDM practice summarized here focus on 36 resources that included clearly stated recommendations. Four other resources provided practical advice in the form of case studies or examples of SFDM. All are included in the discussion of practical recommendations below.

The advice about how to support decision-making fell into two clusters. A core cluster of commonly included forms of support covered: ensuring that information is provided in a format that is accessible for the person (30 of 36 resources; 88.2 per cent); facilitating the person’s ability to communicate, for example, using communication tools or interpreting body language (28; 77.8 per cent); involving others, for example, people who know the person well or relevant professionals (26; 72.2 per cent); the timing of the decision, for example, considering when the person will be most alert or whether the decision can be delayed (19; 52.8 per cent); and thinking about the best environment for the person to make this decision, for example, where they will feel most comfortable or be free of distractions (19; 52.8 per cent).

All these forms of support are included in the MCA Code^61^ and the guidance from NICE^62^ and Paradigm,^63^ though in the NICE guidance, support relating to the environment only features in the definition of ‘practicable steps’^64^ rather than the main section on supporting decision-making.

A cluster of less common practical recommendations was found in 10 or fewer resources. These forms of support included: allowing time for reflection (10; 27.8 per cent); getting to know the person (6; 16.7 per cent); education or training, for example, in the context of decisions about sexual relationships (5; 13.9 per cent); consulting the person about the support they want (4; 11.1 per cent); really listening (4; 11.1 per cent); treating a relevant medical condition, for example, medical treatment or psychological therapy (4; 11.1 per cent); taking a written record of the support provided (4; 11.1 per cent); being mindful that others may undermine decision-making, for example, references to coercion or undue influence (3; 8.3 per cent); practice with the supporter, for example, doing the weekly shopping together (3; 8.3 per cent); revisiting a decision (3; 8.3 per cent); planning how decisions are made (2; 5.6 per cent); getting the right person as the supporter (1; 2.7 per cent); trying options out (1; 2.7 per cent); empathy for decision-making difficulties (1; 2.7 per cent); breaking down the decision (1; 2.7 per cent); addressing any negative effects of medication (1; 2.7 per cent); and considering the wider context of the decision (1; 2.7 per cent).

It was noteworthy that the recommendations to seek input from the decision-maker about the support they want, and to take a written record, are included in the NICE guidance^65^ but were found in few other resources. NICE guidance is generally accorded special legal significance in the hierarchy of clinical guidelines, in part due to the substantial development process that is generally involved.^66^ The authority of NICE guidance in the UK indicates the legal importance of these elements of SFDM in England, raising a question about their absence from resources published after 2018.

We note also that some guidance referred to practical mechanisms for ensuring that the decision-maker has the support they want and need, for example, financial passports (2 documents), health passports (1), communication passports (1), health action plans (1), or a workbook for planning support (1).

### E. Legal foundations

Almost all the guidance (38; 95 per cent) cited the MCA, and this was often the only law referred to in the relevant sections of the resources. Some also referred to other legislation, including the Care Act 2014 (6; 15 per cent), Children and Families Act 2014 (2; 5 per cent), Sexual Offences Act 2003 (1; 2.5 per cent), and Equality Act 2010 (1; 2.5 per cent).

Only four resources referred to the CRPD (10 per cent), one being the guidance from Paradigm.^67^ The other three were guidance published by the Department of Health for independent advocates, on Providing Independent Advocacy under the Care Act’;^68^ an output of an international collaboration involving the English organization, CHANGE, on ‘Good and Promising Practice’ for those delivering training about Article 12^69^; and an MCA ‘Ethics Toolkit’ produced by the British Medical Association.^70^ The previous version of the British Medical Association’s MCA toolkit did not cite the CRPD.^71^ Publication of the current toolkit therefore marks a potentially significant development in thinking about SFDM and its legal foundations in England.

## V. DISCUSSION

The review found that while many organizations in England recommend using or themselves use three primary sources of guidance—the MCA Code^72^ and documents produced by NICE^73^ and Paradigm^74^—some have produced their own. The presence of local or in-house guidance alongside national guidance is also found in other contexts.^75^ The identified local or in-house SFDM guidance tended to be narrower in scope when compared to the three primary pieces of guidance, in terms of intended audience, the decision-maker, or the kinds of decisions being supported. This may indicate a view at the local level, that the primary sources of guidance do not provide sufficiently fine-grained or relevant advice for the implementation of SFDM in many concrete situations. This suggestion resonates with a study of clinical guidance for paediatric asthma in the UK and the Netherlands, which concluded that one ‘driving factor in the widespread development and use of local guidelines … may be the inability of national guidance to achieve sufficient clarity to be used effectively by clinicians’.^76^

This finding points to the potential value of further developing a primary source of guidance to better reflect the multifaceted nature of SFDM in practice. The findings discussed below point to the importance of recognizing the distinctive support needs linked to particular disabilities and conditions; the different barriers to SFDM associated with different kinds of decision and kinds of relationship between supporter and decision-maker; and the need for tailored forms of advice for decision-makers, support providers, and third parties. Presenting a more nuanced account of SFDM in a widely used and authoritative resource, or set of resources, would minimize the need for policy-makers or practitioners to piece together the advice they need at the local level. However, any further development of resources must be mindful that healthcare staff report feeling ‘overwhelmed’ by the volume of guidance relevant to their practice,^77^ and that this is said to undermine its usefulness.^78^

The following subsections discuss the findings set out in the results section.

### A. Audience

The review’s identification of separate guidance for decision-makers, support providers, and third parties is also seen in other jurisdictions. In New South Wales, Australia, for example, separate handbooks have been produced for decision-makers, supporters, and facilitators, who play a role that involves being a mentor to decision-makers and supporters.^79^ The handbook for decision-makers outlines their rights, responsibilities, and what they can expect, whereas the documents aimed at supporters and facilitators outline their respective roles, duties, and the challenges they might face.

Guidance for facilitators was not identified in this review, indicating that this role is not a prominent feature of SFDM as it is currently practiced in England. This highlights a key difference in the implementation of SFDM in England compared to Australian models, in which facilitators play a central role.^80^ Canadian guidance also refers to the role of planning facilitators in delivering ‘core functions’ of community-led SFDM initiatives,^81^ and the role of facilitator is recognized to some extent in US initiatives.^82^ This finding prompts a question about whether the role of facilitator is a dimension of SFDM practice that should be developed in England.

Most of the guidance identified in this review was aimed at support providers. While the MCA Code^83^ and documents produced by NICE^84^ and Paradigm^85^ were not aimed at providers in specific roles, many other documents were, for example, for medical professionals or family members. One potential advantage of guidance that is sensitive to the supporter’s role is indicated by an Australian study that found the most common barrier to SFDM was, ‘tensions associated with the role of the supporter and consequent conflict with others involved in the decision-maker’s life’.^86^ This indicates the importance of guidance acknowledging that the barriers faced by supporters may depend on their relationship with the decision-maker, for example, whether they are a parent, child, friend, medical professional, or paid advocate.

A small number of the identified resources (4; 10 per cent) were aimed at decision-makers (people being supported), with three designed for any adult who may need support in decision-making and one for adults with learning disabilities. Guidance of this kind seems crucial for upholding the values that motivate SFDM, such as inclusion and independence. Empirical work has underlined the importance of informing mental health service users of their rights and the decision-making support they should expect.^87^ The guidance designed for decision-makers identified in this review provided distinctive advice on whose support the decision-maker can enlist to make important decisions,^88^ their right to open a bank account, and how they should expect to be treated during the process.^89^

A similar proportion of guidance in the sample (3; 7.5 per cent) was aimed at third parties who may be involved in enacting supported decisions, for example, banking or healthcare staff. Third parties play a crucial role in the recognition of decisions made with support, and they require distinctive advice about what obligation they have to involve a supporter, and recognizing when supported decisions should, and should not, be acted upon. A Canadian study found that third parties were ‘often not comfortable with’ SFDM arrangements, and the study recommended that materials be developed specifically for third-party healthcare providers and financial institutions.^90^

Overall, the findings regarding audience suggest that SFDM in England might be promoted through the development of authoritative guidance for decision-makers and third parties. The question of whether the facilitator role in SFDM is being overlooked in this jurisdiction should also be explored.

### B. Decision-maker (person being supported)

While the majority of the identified resources, including the MCA Code,^91^ NICE,^92^ and Paradigm^93^ documents, provided guidance relevant for anyone in need of SFDM, approximately one-third of the resources (32.5 per cent) were about support for people with specified disabilities or medical conditions, the most common being learning disabilities.

One potential justification for SFDM guidance focused on decision-makers with particular disabilities or conditions comes from research that suggests different support strategies may be useful for different disabilities or conditions.^94^ However, for many people needing support, the difficulties they face will be connected to more than one disability or condition. For example, there are high rates of mental ill-health among people with learning disabilities,^95^ dementia,^96^ and autistic people.^97^ The MCA Code recognizes that some organizations have developed SFDM materials for ‘specific conditions, such as Alzheimer’s disease or profound learning disability’.^98^ However, the inclusion of some of this tailored advice in a primary source of guidance would, arguably, make these resources more useful in practice.

The prominence in the sample of guidance about supporting people with a learning disability is consistent with a significant focus on this group in SFDM research.^99^ However, there is a growing body of relevant research on decision-making in other groups, including people with dementia,^100^ acquired brain injury,^101^ autistic people,^102^ and those with mental health conditions.^103^ This indicates the need for guidance to draw widely on relevant research in connection with a range of disabilities and conditions.

It was noteworthy that the review did not identify any guidance with a dedicated or primary focus on SFDM in the context of mental ill health, while other jurisdictions have seen developments in this area.^104^ The Mental Welfare Commission for Scotland has a detailed guide on SFDM, which includes mental health conditions.^105^ In Victoria, Australia, the Mental Health and Wellbeing Act 2022 Handbook elaborates on the legislation’s ‘Supported decision making principle’ referring to support measures around information provision, communication, and advance statements.^106^ Predating this guidance, the Victorian branch of the Royal Australian and New Zealand College of Psychiatrists published a position paper on ‘Enabling supported decision making’.^107^ More recently, the Mental Health Foundation of New Zealand published a position statement on ‘Embedding supported decision-making across Aotearoa’s mental health system’.^108^ However, recent guidance from NHS England on acute inpatient mental health care for adults includes ‘Personalised care, including shared decision-making’^109^ as one of its four key principles but does not focus on supported decision-making among its comprehensive recommendations.

Research on SFDM in connection with mental ill health has highlighted some challenges in this area, particularly concerning the trust relationships that are necessary.^110^ Nonetheless, it appears that the implementation of SFDM in mental health care is moving ahead in other jurisdictions.^111^

### C. Decisions being supported

While most of the identified guidance, including the MCA Code^112^ and documents from NICE^113^ and Paradigm,^114^ took a generic approach in terms of the decisions being supported, approximately a quarter of the sample (27.5 per cent) focused on narrower spheres of decision-making such as healthcare or financial decisions.

One argument in favour of the narrower approach might appeal to the idea that different kinds of decisions require different forms of support. Research by Harding and Taşcıoğlu in England and Wales found that legally significant decisions, in contrast with more everyday decisions, often involve a more complex decision-making process.^115^ Care professionals in their research identified three main approaches to supporting everyday decisions, involving narrowing down choices, providing context, and establishing structures to help execute decisions.^116^ In contrast, complex decisions were found to require more advanced strategies such as participation in goal setting, exploring alternative choices, making information accessible, creating a power of attorney, advance planning, and involving an independent advocate.^117^ The research concluded that the advice on SFDM in the MCA Code^118^ is not sufficient to support the full range of personal decisions.^119^

A more decision-specific approach to providing guidance is also able to highlight the barriers to SFDM associated with particular types of decisions. For example, health and social care practitioners cite concerns about personal liability when supporting someone to make a decision that could result in injury or abuse.^120^ Harding and Taşcıoğlu found that decisions about intimate relationships and sexuality were not well supported due to apprehensions about abuse.^121^

This research points to the importance of including decision-specific nuance in SFDM guidance, including advice on how to handle the risks associated with particular kinds of decisions. In guidance that aims to provide a widely applicable model of SFDM, recommendations designed for any decision will be a useful starting point. However, more fine-grained advice appears to be crucial for ensuring that the support provided is appropriate for the decision and for addressing the barriers that may be present.

### D. Recommendations for practice: the elements of support for decision-making

A key observation about the practical advice found in the sample was its variability. Among the cluster of ways to support decision-making that were commonly included, only three—making information accessible, facilitating communication, and involving others—were found in a significant majority of the guidance. All other recommendations were found in approximately half or fewer of the resources. It was noted above that some elements included in the NICE guidance, for example, seeking input from the person about the support they want, were found in a few of the other identified documents.^122^ This raises a question about the evidence or authority on which SFDM guidance is being based, and is a finding that resonates with clinical practice guidelines research in the UK. Koldeweij and colleagues have raised concerns about variability between locally produced clinical guidance documents and divergence from NICE guidelines.^123^ The authors acknowledge that some adaptation of national clinical guidelines may be necessary in locally produced documents due to factors such as the needs, resources, and expertise available in the local context. Such factors may also play a part in explaining the variability of advice found in the SFDM guidance, along with differences related to, for example, a focus on a particular disability or condition in some of the identified resources. Nonetheless, Koldeweij and others caution that, ‘variability in the content and quality of local guidelines may contribute to variation of care more broadly and [may] potentially undermine healthcare quality’,^124^ and this concern also seems relevant in the context of SFDM.

Overall, the practical advice found in the sample reflected several strategies for supporting complex decisions reported by care professionals in England and Wales in the work of Harding and Taşcıoğlu, particularly around making information accessible and involving others.^125^ However, other dimensions of SFDM reported by care professionals, such as participation in goal setting and exploring alternative choices, were not prominent in the identified guidance. This raises a question about whether important elements of SFDM are not included in the existing guidance, a concern previously identified by Harding and Taşcıoğlu in relation to the MCA Code.^126^

The La Trobe SFDM Practice Framework, developed by Bigby and Douglas,^127^ provides a useful international reference point for reflecting on the advice found in the sample. The framework is an established model for SFDM in Australia, based on a body of empirical work.^128^ It takes a multi-layered approach involving three guiding principles, seven steps for supporting any decision, and five clusters of support strategies that can be used when appropriate. The principles emphasize, ‘Commitment: to the person and their rights’, ‘Orchestration: of others involved in the person’s life’, and ‘Reflection and Review: on your own values and influence in support’.^129^ The steps move through, ‘Knowing the person’, ‘Identifying and describing the decision’, ‘Understanding the person’s will and preference for the decision’, ‘Refining the decision and taking account of constraints’, ‘Deciding whether a self-generated, shared or substituted decision is to be made’, ‘Reaching the decision and associated decisions’, to ‘Implementing the decision and seeking out advocates if necessary’.^130^ Lastly, the clusters of strategies concern, ‘Attention to communication’, ‘Education about consequences and practicalities’, ‘Listening and engaging to ensure that all options are considered’, ‘Creating opportunities’, and ‘Breaking things down’.^131^

This model provides a richer account of how to implement SFDM than was found in any of the guidance identified in this review. Its foundation in the CRPD^132^ also distinguishes this framework from much of the identified English guidance.^133^

Four of the framework’s five clusters of practical strategy were found in the English guidance: those about communication, education, listening, and breaking things down. The strategy that was not well reflected in the identified documents concerned creating opportunities. In the La Trobe framework, this strategy is described as including, ‘Active reframing that invites participation—providing a sounding board—acknowledging low expectations and building confidence—testing options—introducing and nurturing the seeds of ideas—bringing in others to trail a situation—creating distance to enable greater autonomy’.^134^ One observation about these forms of support is that some, for example, building confidence and nurturing the seeds of ideas, seem to require a long-term support relationship. Department of Health guidance for Independent Advocates endorses this way of envisaging SFDM in England, stating that, ‘Supported decision making is about much more than helping people to make one off decisions, or providing support during a mental capacity assessment. It is about a holistic system of support that places the person at the centre of decision making’.^135^ Yet many of the identified documents envisage SFDM in a more limited way that is linked to individual decisions and capacity assessments.

A related observation concerns the purpose of SFDM, as reflected in the sample. A primary focus on providing accessible information and facilitating communication sees the purpose of support as enabling decision-making within the scope of the person’s existing abilities and limitations. However, among the less commonly found recommendations was advice that understands the purpose of support in a more developmental way. For example, four documents included decision-relevant education or training as a form of support, and three included the treatment (medical or psychological) of a relevant condition. Practicing decision-making with the supporter also featured in three documents. Guidance that includes these forms of support sees the decision-maker as having the potential to grow their capacities for independent decision-making agency. This perspective is illustrated in a document produced by the British Psychological Society, which included a case study that described using role play, with the outcome that, ‘Over time, observation indicated that [the person] was able to utilise the skills in novel situations without prompting’.^136^

Although these developmental elements of SFDM were not commonly found in the sample documents, some are included in the MCA Code and NICE guidance.^137^ This indicates the importance, from a legal perspective in England, of understanding support as a developmental process, not merely one that works within the decision-maker’s existing scope for exercising decision-making agency with support.

### E. Legal foundations

Most of the identified resources referred primarily, or solely, to the MCA, while only four referred to the CRPD. This finding resonates with Harding and Taşcıoğlu’s research, which found that while principle 3 of the MCA, which concerns SFDM, is ‘well embedded in the professional awareness’ of care practitioners in England and Wales, ‘few had heard of’ the CRPD.^138^ Of the three primary sources of guidance, only the Paradigm document refers to the CRPD, stating that, ‘Supported Decision-Making is a basic human right that is highlighted in the “UN Convention on the Rights of Persons with Disabilities” and is part of the Mental Capacity Act 2005 and The Care Act 2014’.^139^ The linking of SFDM to the CRPD in recent guidance from the British Medical Association seems likely to mark a significant development in this area, due to the legal weight it can be given.^140^ Nonetheless, its recommendations are framed in terms of how ‘doctors can draw on aspects of the CRPD’s supported decision-making approach to complement their obligations under the support principle in the MCA’.^141^

The dominance of the MCA as the legal foundation of SFDM in the identified guidance is no doubt explained by the legally binding status of the MCA in England, not shared by the CRPD, along with the apparent tensions between the MCA and CRPD conceptions of SFDM.^142^ In its initial report on the United Kingdom of Great Britain and Northern Ireland, the UN treaty body for the CRPD expressed concerns about, ‘legislation restricting the legal capacity of persons with disabilities on the basis of actual or perceived impairment … the prevalence of substituted decision-making in legislation and practice, and the lack of full recognition of the right to individualised supported decision-making that fully respects the autonomy, will and preferences of persons with disabilities’.^143^ These observations raise questions about the prospect of realizing a CRPD-aligned approach to SFDM in England, due to the presence of MCA.

The framing of SFDM primarily in connection with the MCA, as a means to enable mental capacity, provides an indication of how those involved in SFDM in England are generally encouraged to understand its purpose. The focus is on facilitating the person’s decision-making abilities, rather than the legal recognition of their will and preferences. This difference has significant implications because, for example, a focus on will and preferences might allow the person being supported to retain recognition as the decision-maker despite one or more dimensions of mental capacity being provided by the supporter. This model seems incompatible with the understanding of SFDM linked to the MCA, where the mental capacities needed to make a decision must be attributed to the person being supported, or they will not be recognized as the decision-maker. On this understanding, the limits of SFDM are set by the extent to which the person’s own mental capacities can be enabled. If *they* are unable to satisfy the mental capacity requirements, the possibilities for SFDM are exhausted, and the decision will be made by others in their best interests.^144^

Given these tensions, it may be that policymakers have tended to take an approach that avoids this complexity in order to provide clear recommendations. However, the UK’s commitment to the CRPD places an obligation on policymakers to explore whether practice can move towards the understanding of SFDM envisaged in the CRPD. Recommending such an approach, the Department of Health guidance acknowledges that, ‘Advocates are obliged to work within the MCA framework under domestic law’, but that ‘the MCA and the Care Act contain important tools that can be used by advocates to promote the empowering ethos of the CRPD’, and also that the ‘CRPD pushes us to move toward a new framework where people are supported to be their own decision-makers’.^145^

The recent British Medical Association guidance takes a more cautious approach, indicating that where there ‘may’^146^ be tensions between the MCA and CRPD around SFDM, a resolution is already in play. Its section on ‘supported decision making’ follows a section on best interests decisions and observes that a will and preferences approach ‘now guides decisions in the Court of Protection’.^147^ It goes on to explain that the focus is ‘increasingly on determining what the individual would want—and consider to be in their best interests—in the circumstances, rather than what others believe objectively to be in their best interests’.^148^ On this approach, the support required by Article 12 may be realized in the context of some substituted decisions under the MCA, with the crucial element of a CRPD-aligned approach to SFDM understood to be its emphasis on foregrounding the person’s perspective, rather than who makes the decision.

This way of envisaging the implementation of Article 12 has been recognized in the legal commentary, with Bartlett observing that although this arrangement would ‘be triggered by incapacity, and to that extent would be inconsistent with the CRPD as the Committee articulates it, … it might be coherently argued that this sort of agency relationship would be within the spirit of the CRPD’.^149^ This form of resolution nonetheless raises concerns that the ‘transformative potential of the CRPD will not be realised’ if its implementation replicates existing binaries such as the ‘distinction between capacity and incapacity’.^150^ Harding and Taşcıoğlu argue on the basis of their work, that ‘the MCA’s decision-specific approach, underpinned by the functional assessment of capacity, allows and indeed facilitates the drive towards substitute decision-making for more complex matters’.^151^ Similarly, the Australian Royal Commission report rejects capacity-based models on the basis that they perpetuate the idea that SFDM is ‘something that is done “up to a point”’ demarcated by a mental capacity threshold.^152^

Another consequence of this proposed resolution becomes clear in the BMA guidance. Both the Australian Royal Commission report^153^ and BMA guidance reject a strict binary between supported and substituted decision-making, with substituted decisions counting as instances of SFDM in alignment with the CRPD when they promote the legal recognition of the person’s will and preferences. Evidence gathered by Bigby and colleagues suggests that this way of understanding SFDM may better reflect the reality of having and providing support in decision-making.^154^ However, the presence of the MCA in England and Wales means that its implementation in this jurisdiction generates a dualism in the purpose and practice of support. As described in the BMA guidance, where a person may retain mental capacity, SFDM is about enabling their decision-making abilities through practices including the involvement of an advocate, communication support, and considering the timing of the decision or the impact of medication.^155^ In situations where a person is unable to meet this standard, recommendations for SFDM include exploring with the person what support may be helpful, identifying the person’s wishes and feelings, maximizing the person’s involvement, and facilitating the involvement of others who are close to the person.^156^ On this side of the capacity threshold, the purpose of SFDM is less clear, but the focus has shifted from supporting the person’s decision-making abilities to involving the person and seeking an understanding of the decision from their perspective. This dualism is not a feature of the Australian Royal Commission report model, in which capacity-based approaches are rejected in favour of a will and preferences approach across all SFDM.^157^

The question that comes into focus is whether this dualism in the purpose and practice of SFDM is problematic, from a practical, legal, or ethical perspective. This question adds to, and provides a more concrete context for, the above concerns raised by others, and may also be relevant in other jurisdictions with similar mental capacity law.

## VI. CONCLUSION

The aim of this pragmatic review was to provide insight into how the practice of SFDM is developing in England, as reflected in a sample of relevant guidance. A key subsidiary aim was to explore the legal foundation of the guidance given the identified tensions and uncertainties around the understandings of SFDM linked to the MCA and CRPD. It was also anticipated that the findings may identify gaps or weaknesses in current guidance, indicating areas for the development of SFDM practice in England.

The review only identified limited guidance for decision-makers (those being supported), or for third parties. In contrast with some other jurisdictions, no guidance for the role of support facilitators or guidance specifically relating to decisions by people with mental health diagnoses, was identified. These are therefore recommended as focal points for those working on the development of SFDM policy and practice in England.

Regarding recommendations for the practice of SFDM, the review found a great deal of variability. Where elements of the MCA Code or NICE guidance were not included in other identified documents, a question is raised about the basis and authority of that guidance. However, some of the variability in the sample likely reflects relevant differences between the many possible contexts for SFDM. It may also be linked to a lack of consensus or clarity about fundamental issues concerning its proper scope and purpose. To what degree is SFDM about enabling the person’s decision-making capacities or realizing their will and preferences? Is it appropriate for supporters to play a substantial role in the deliberative process, in effect providing some decision-making abilities that may be necessary? Does SFDM extend to more developmental forms of support?

Some of these unsettled questions are linked to the issue of legal foundations. Most of the identified guidance reflected an exclusive alignment with an understanding of SFDM linked to the MCA, as a means to enable the decision-maker’s mental capacity. This finding is understandable due to the legally binding requirements of the MCA in England, combined with the apparent tensions between the MCA and CRPD approaches to SFDM. Nonetheless, the UK’s commitment to the CRPD places an obligation on policymakers to consider how SFDM practice under the MCA might be developed in alignment with the CRPD. Some recent developments propose a resolution that rejects a strict binary between substituted and supported decisions, envisaging a CRPD-aligned approach to SFDM realized in the context of some substituted decisions. However, applying this approach in England and Wales generates a dualistic model in which SFDM diverges, in purpose and practice, on either side of the MCA’s capacity threshold. This calls for evaluation from a practical, legal, and ethical perspective.

This complexity, in the diversity of SFDM contexts and uncertainties linked to the legal foundations of SFDM, presents a challenge for generating guidance. The analysis suggests that the currently fragmented state of SFDM guidance in England could be addressed through the further development of an authoritative source of guidance to present a more multifaceted model of SFDM. Important dimensions would include insights from SFDM research about the support needs associated with certain decisions, disabilities, and conditions, as well as the barriers associated with particular kinds of decision and support relationship.

It may also be helpful for guidance to more widely acknowledge tensions in the legal foundations of SFDM, as these may resonate with dilemmas faced in practice. The British Association of Social Workers recommends that those working with adults with intellectual disabilities should, ‘[k]now about the historical, theoretical, and ethical contexts of mental capacity practice, supported decision-making, and human rights’.^158^ To be meaningful, what’s required is not just referring to the empowering ethos of the CRPD, but recognizing the challenging questions it raises about SFDM.

## Footnotes

1 T Stainton, ‘Supported Decision-making in Canada: Principles, Policy, and Practice’ (2016) 3 Research and Practice in Intellectual and Developmental Disabilities <https://www.tandfonline.com/doi/abs/10.1080/23297018.2015.1063447> accessed 15 May 2025; G Davidson and others, ‘Supported Decision-making—Experiences, Approaches and Preferences’ (Queen’s University Belfast 2018) <https://pureadmin.qub.ac.uk/ws/portalfiles/portal/162865093/Supported_decision_making_standard_report_online.pdf> accessed 14 May 2025; C McKay and others, ‘Scottish Mental Health Law Review: Final Report’ (Scottish Government 2022) <https://webarchive.nrscotland.gov.uk/20230327160310/https:/cms.mentalhealthlawreview.scot/wp-content/uploads/2022/09/SMHLR-FINAL-Report-.pdf> accessed 14 May 2025; C Bigby and others, ‘Diversity, Dignity, Equity and Best Practice: A Framework for Supported Decision-making’ (Royal Commission into Violence, Abuse, Neglect and Exploitation of People with Disability, 2023) <https://disability.royalcommission.gov.au/publications/diversity-dignity-equity-and-best-practice-framework-supported-decision-making> accessed 13 May 2025.

2 MCA, s 1(3)); Care Act 2014, s 1(2)(d); ‘An individual may be unable to request an assessment [for care and support] or may struggle to express their needs. The local authority must in these situations carry out supported decision making, helping the person to be as involved as possible in the assessment, and must carry out a capacity assessment.’ Department of Health and Social Care, *Care and Support Statutory Guidance* (2025) s 6.11.

3 J Beadle-Brown, ‘Supported Decision-making in the United Kingdom: Lessons for Future Success (2015) 2 Research and Practice in Intellectual and Developmental Disabilities 17; R Harding and E Taşcıoğlu, ‘Everyday Decisions Project Report: Supporting Legal Capacity Through Care, Support and Empowerment’ (University of Birmingham 2017) <https://legalcapacity.org.uk/wp-content/uploads/2017/12/Everyday_Decisions_Project_Report.pdf> accessed 21 May 2025; R Harding and E Taşcıoğlu, ‘Supported Decision-Making from Theory to Practice: Implementing the Right to Enjoy Legal Capacity’ (2018) 8 Societies <https://www.mdpi.com/2075-4698/8/2/25> accessed 14 May 2025.

4 AR Gagliardi and others, ‘How Can We Improve Guideline Use? A Conceptual Framework of Implementability’ (2011) 6 Implementation Science <https://implementationscience.biomedcentral.com/articles/10.1186/1748-5908-6-26> accessed 14 May 2025.

5 SH Woolf and others, ‘Clinical Guidelines: Potential Benefits, Limitations, and Harms of Clinical Guidelines’ (1999) 318 British Medical Journal 527.

6 J Tingle, ‘The Importance of Keeping up to Date with Clinical Guidelines and Protocols’ (2023) 32 British Journal of Nursing 266.

7 D Metcalf, C Pitkeathly and J Herring, ‘“Advice, Not Orders”? The Evolving Legal Status of Clinical Guidelines’ (2021) 47 Journal of Medical Ethics <https://jme.bmj.com/content/47/12/e78> accessed 15 May 2025; Tingle (n 6).

8 AM Turner and others, ‘Modelling Public Health Interventions for Improved Access to the Grey Literature’ (2005) 93 Journal of the Medical Library Association 487; K Godin and others, ‘Applying Systematic Review Search Methods to the Grey Literature: A Case Study Examining Guidelines for School-based Breakfast Programs in Canada’ (2015) 4 Systematic reviews <https://systematicreviewsjournal.biomedcentral.com/articles/10.1186/s13643-015-0125-0> accessed 14 May 2025; J Adams and others, ‘Searching and Synthesising “grey literature” and “grey information” in Public Health: Critical Reflections on Three Case Studies’ (2016) 5 Systematic Reviews <https://systematicreviewsjournal.biomedcentral.com/articles/10.1186/s13643-016-0337-y> accessed 13 May 2025.

9 s 1(3).

10
*A Local Authority v JB (Rev1)* [2021] UKSC 52, s 118.

11 National Institute for Health and Care Excellence, ‘Decision-making and Mental Capacity’ (NICE guideline, NG108, 2018) s 1.2 <https://www.nice.org.uk/guidance/ng108> accessed 15 May 2025.

12 art 12.3.

13 Committee on the Rights of Persons with Disabilities, ‘General Comment No. 1—Article 12: Equal Recognition Before the Law’ (United Nations, CRPD/C/GC/1, 2014) para 29.

14 Australian Law Reform Commission, *Equality, Capacity and Disability in Commonwealth laws: final report* (ALRC report 124, 2014); McKay and others (n 1). See Ruck Keene and colleagues for an account of how art 12 of the CRPD has been used to challenge mental capacity law in England and Wales; A Ruck Keene and others, ‘Mental Capacity—Why Look for a Paradigm Shift?’ (2023) 31 Medical Law Review 340.

15 ‘The concept of mental capacity is highly controversial in and of itself. Mental capacity is not, as is commonly presented, an objective, scientific and naturally occurring phenomenon. Mental capacity is contingent on social and political contexts, as are the disciplines, professions and practices which play a dominant role in assessing mental capacity.’ Committee on the Rights of Persons with Disabilities (n 13) para 14; see Ruck Keene and others (n 14) for a critique of this position.

16 P Gooding, ‘Supported Decision-Making: A Rights-Based Disability Concept and its Implications for Mental Health Law’ (2013) 20 Psychiatry, Psychology and Law 431; M Browning and others, ‘Supported Decision Making: Understanding How its Conceptual Link to Legal Capacity is Influencing the Development of Practice’ (2014) Research and Practice in Intellectual and Developmental Disabilities 1 34; T Carney and others, ‘Realising “will, preferences and rights”: Reconciling Differences on Best Practice Support for Decision-making?’ (2019) 28 Griffith Law Review 357; G Szmukler, ‘“Capacity”, “best interests”, “will and preferences” and the UN Convention on the Rights of Persons with Disabilities’ (2019) 18 World Psychiatry 34; P Bartlett, ‘At the Interface Between Paradigms: English Mental Capacity Law and the CRPD’ (2020) 11 Frontiers in Psychiatry <https://www.frontiersin.org/journals/psychiatry/articles/10.3389/fpsyt.2020.570735/full> accessed 13 May 2025; J Craigie, ‘Conceptualising “Undue Influence” in Decision-Making Support for People with Mental Disabilities’ (2021) 29 Medical Law Review 48.

17 McKay and others (n 1) 246.

18 Select Committee on the Mental Capacity Act 2005, ‘Mental Capacity Act 2005: Post-Legislative Scrutiny’ (House of Lords, HL paper 139, 2014) p 41.

19 S Goldman, ‘Care England Mental Capacity Act Implementation Survey: Report’ (Care England, 2016) <http://www.careengland.org.uk/sites/careengland/files/Care%20England%20MCA%20Implementation%20Survey%20report_0.pdf> accessed 14 May 2025.

20 R Kokanović and others, ‘Supported Decision-making from the Perspectives of Mental Health Service users, Family Members Supporting them and Mental Health Practitioners’ (2018) 52 Australian and New Zealand Journal of Psychiatry 826; L Penzenstadler, A Molodynski and Y Khazaal, ‘Supported Decision-Making for People with Mental Health Disorders in Clinical Practice: A Systematic Review’ (2019) 24 International Journal of Psychiatry in Clinical Practice 3.

21 M Browning, ‘Developing an Understanding of Supported Decision-Making Practice in Canada: The Experiences of People with Intellectual Disabilities and Their Supporters’ (PhD Thesis, La Trobe University 2018) p 184 <https://opal.latrobe.edu.au/articles/thesis/Developing_an_understanding_of_supported_decision-making_practice_in_Canada_the_experiences_of_people_with_intellectual_disabilities_and_their_supporters/21857775?file=38787954> accessed 13 May 2025.

22 A Dhanda, ‘Legal Capacity in the Disability Rights CRPD: Stranglehold of the Past or Lodestar for the Future?’ (2007) 34 Syracuse J Int’l L & Com 429, 431; Committee on the Rights of Persons with Disabilities (n 13) para 15.

23 L Haworth, *Autonomy: An Essay in Philosophical Psychology and Ethics* (Yale University Press 1986) chap 2; J Pugh, *Autonomy, Rationality, and Contemporary Bioethics* (OUP 2020) chap 2.

24 H Frankfurt, ‘Freedom of the Will and the Concept of a Person’ in H Frankfurt (ed), *The Importance of What We Care About* (CUP 1987). See also Haworth (n 23) 20, Chap 3.

25 R Dworkin, *Life’s Dominion: An Argument about Abortion, Euthanasia, and Individual Freedom* (Vintage Books 1994) 230.

26 A Jaworska, ‘Respecting the Margins of Agency: Alzheimer’s Patients and the Capacity to Value’ (1999) 28 Philosophy and Public Affairs 82.

27 J Doris, *Talking to Ourselves: Reflection, Ignorance, and Agency* (OUP 2015) X and 40; emphasis is in the original.

28 ibid 33. Doris does seem to accept, rather than challenge, standard practices of withholding the recognition of agency in connection with mental disability or mental ill health (Doris (n 27) 34 and 39). For example, Doris writes that, ‘Inability to recognize which circumstances are conducive to the expression of one’s values would presumably impair one’s ability to act in ways expressing one’s values; such a cognitive disability could be a disability afflicting the exercise of agency’. (Doris (n 27) 106; see also 34–35). However, the separation of agency from the exercise of reflection and reasoning in this account does open the door to conversations about how people ‘with a range of cognitive attainments may exercise agency’ (Doris (n 27) 40).

29 For example, the Committee on the Rights of Persons with Disabilities holds that, ‘All people … have legal standing and legal agency simply by virtue of being human’ (n 13) para 14.

30 Committee on the Rights of Persons with Disabilities (n 13) paras 13, 24, 25.

31 ibid para 17.

32 ibid para 13.

33 Bigby and others (n 1) 22.

34 ibid.

35 ibid.

36 ibid 23.

37 SFDM is a term used by others. C Bigby and J Douglas, ‘Support for Decision Making: A Practice Framework’ (La Trobe University 2016) <https://opal.latrobe.edu.au/articles/report/Support_for_decision_making_-_a_practice_framework/22240162?file=39526903> accessed 22 May 2025. For an online learning version of the resource see: C Bigby, J Douglas and S Vassallo, ‘The La Trobe Support for Decision Making Practice Framework’ (La Trobe University 2019) <https://www.supportfordecisionmakingresource.com.au/> accessed 2 July 2025.

38 Committee on the Rights of Persons with Disabilities (n 13) paras 3, 28.

39 ibid, para 17.

40 Godin and others (n 8).

41 Turner and others (n 8); Godin and others (n 8); Adams and others (n 8).

42 S Pathare and L Shields, ‘Supported Decision-Making for Persons with Mental Illness: A Review’ (2012) 34 Public Health Reviews <https://publichealthreviews.biomedcentral.com/articles/10.1007/BF03391683> accessed 15 May 2025; PM Gooding and MB Simmons, ‘Spot the Difference: Shared Decision-making and Supported Decision-making in Mental Health’ (2017) 34 Irish Journal of Psychological Medicine 275. See also: National Institute for Health and Care Excellence, ‘Shared Decision Making’ (NG197, National Institute for Health and Care Excellence 2021) <https://www.nice.org.uk/guidance/ng197>.

43 Search terms included: ‘Mental Health/learning disability/advocacy organisations’, ‘advocacy charity’, ‘Mental Health/learning difficulties/brain injury/Alzheimer’s charity’, ‘Mental Health/learning difficulties/brain injury/Alzheimer’s organisations’, ‘carer organisations’, ‘decision support guide’, ‘decision support guide’ and ‘Mental Capacity Act guide’.

44 Godin and others (n 8).

45 eg, a document produced by the Royal College of Surgeons was excluded on the basis that it included only standard guidance for an informed consent process. Royal College of Surgeons, *Consent: Supported decision-making, A Guide to Good Practice* (Royal College of Surgeons 2018) <https://www.rcseng.ac.uk/standards-and-research/standards-and-guidance/good-practice-guides/consent/> accessed 15 May 2025. An online resource hub providing practical tools and information that may be useful in providing SFDM was excluded on the basis that it did not constitute a guidance document providing recommendations on how to support decision-making.

46 Department for Constitutional Affairs, *Mental Capacity Act 2005 Code of Practice* (The Stationary Office 2007).

47 National Institute for Health and Care Excellence (n 11).

48 K Fulton, K Woodley and H Sanderson, ‘Supported Decision-Making: A Guide for Supporters’ (Paradigm 2008); updated in 2023 to become: S Warren and J Giles, ‘A Practical Guide to Supported Decision-Making’ (Paradigm 2023). <https://paradigm-uk.org/wp-content/uploads/2023/01/Print-copy-2023-MASTER-Supported-Decisions-Making-booklet-27-September-A5_Wiro_Bound_SEB.pdf.pdf> accessed 15 May 2025.

49 Warren and Giles (n 48).

50 HM Government, *Mental Capacity Act 2005 Code of Practice including the Liberty Protection Safeguards (Draft)* (Crown Copyright 2022) <https://assets.publishing.service.gov.uk/media/62962d1f8fa8f50395c0a054/draft-mental-capacity-act-code-of-practice.pdf> accessed 14 May 2025.

51 Department for Constitutional Affairs (n 46).

52 ibid.

53 National Institute for Health and Care Excellence (n 11).

54 Warren and Giles (n 48).

55 Department for Constitutional Affairs (n 46).

56 National Institute for Health and Care Excellence (n 11).

57 Warren and Giles (n 48).

58 Department for Constitutional Affairs (n 46).

59 National Institute for Health and Care Excellence (n 11).

60 Warren and Giles (n 48).

61 Department for Constitutional Affairs (n 46).

62 National Institute for Health and Care Excellence (n 11).

63 Warren and Giles (n 48).

64 National Institute for Health and Care Excellence (n 11) 33.

65 ibid s 1.2.1 and 2.1.17.

66 Metcalf, Pitkeathly and Herring (n 6).

67 Warren and Giles (n 48). The previous version of this guide also referred to the CRPD. Fulton, Woodley and Sanderson (n 48).

68 Department of Health, ‘Providing Independent Advocacy under the Care Act’ (The Stationary Office 2014) <https://www.local.gov.uk/sites/default/files/documents/self-study-pack-669.pdf> accessed 23 May 2025.

69 IDEA 12, ‘Good and Promising Practice Guide: Inclusive training about Article 12’ (IDEA 12, 2017) <https://www.idea12.eu/res/archive/001/000228.pdf?seek=1537175066> accessed 21 May 2025.

70 British Medical Association, ‘Ethics Toolkit, Mental Capacity Act—England and Wales’ (British Medical Association, 2024, updated 2025) <https://www.bma.org.uk/media/4z1l3khg/mental-capacity-act-guidance-england-and-wales-updated-feb-2025.pdf> accessed 16 May 2025.

71 British Medical Association, ‘Mental Capacity Act tool kit’ (British Medical Association 2016).

72 Department for Constitutional Affairs (n 46).

73 National Institute for Health and Care Excellence (n 11).

74 Warren and Giles (n 48).

75 Tingle (n 6); C Koldeweij and others, ‘Mind the Gap: Mapping Variation between National and Local Clinical Practice Guidelines for Acute Paediatric Asthma from the United Kingdom and the Netherlands (2022) 17 PLOS One <https://journals.plos.org/plosone/article?id=10.1371/journal.pone.0267445> accessed 14 May.

76 Koldeweij and others (n 75) 11.

77 Tingle (n 6) 226.

78 Metcalf, Pitkeathly and Herring (n 6) 1.

79 Family and Community Services, ‘My Life, my Decision: A Handbook for Decision Makers’ (New South Wales Government 2015a); Family and Community Services, ‘Supported Decision Making: A handbook for facilitators’ (New South Wales Government 2015b); Family and Community Services, ‘Supported Decision Making: A handbook for Supporters’ (New South Wales Government 2015c).

80 See relevant sections of an Australian SFDM pilot evaluation, which states that, ‘The role of facilitator was of key importance and written resources did not reduce the need for this role.’ C Purcal and others, ‘Evaluation of the Supported Decision Making Phase 2 (SDM2) Project: Final Report’ (Social Policy Research Centre, University of New South Wales 2017) 61; see also 11, 19, 35. <https://unsworks.unsw.edu.au/entities/publication/afa221be-9aaf-4266-b3ed-c83e73991d3c> accessed 15 May 2025.

81 Institute for Research and Development on Inclusion and Society, ‘Community-led Initiatives Supporting the Right to Decide’ (Institute for Research and Development on Inclusion and Society 2021) <https://irisinstitute.ca/resource/community-led-initiatives-supporting-the-right-to-decide-a-framework-for-design-and-implementation/> accessed 14 May 2025.

82 E Pell, ‘Supported Decision-Making New York: Evaluation Report of an Intentional Pilot’ (Hunter College/The Research Foundation CUNY 2019) <https://sdmny.org/resources/sdmny-evaluation-report/> accessed 15 May 2025.

83 Department for Constitutional Affairs (n 46).

84 National Institute for Health and Care Excellence (n 11).

85 Warren and Giles (n 48).

86 C Bigby and others, ‘Delivering Decision-making Support to People with Cognitive Disability—What has been Learned from Pilot Programs in Australia from 2010 to 2015’ (2017) 52 Australian Journal of Social Issues 222, 234.

87 Kokanović and others (n 20).

88 H Sanderson and S McStravick, ‘In the Driving Seat: A Workbook to Help me Plan my Support’ (in Control Publications 2007) <https://in-control.org.uk/wp-content/uploads/2020/11/In-the-Driving-seat-aug-07.pdf> accessed 14 May 2025.

89 M Beckford, ‘Making Banking Easier—A Guide Written by Dosh on How to Work with Banks to get What you Need’ (Dosh Financial Advocacy, 2014) < https://www.dosh.org/wp-content/uploads/2016/09/Dosh-banking-guide-2014-Final-v2.pdf> accessed 14 May 2025.

90 K James and L Watts, ‘Understanding the Lived Experiences of Supported Decision-making in Canada: Legal Capacity, Decision-making and Guardianship’ (2014) (Law Commission of Ontario, 2014) 68 <https://www.lco- cdo.org/wp-content/uploads/2014/03/capacity-guardianship-commissioned-paper- ccel.pdf> accessed 22 May 2025.

91 Department for Constitutional Affairs (n 46).

92 National Institute for Health and Care Excellence (n 11).

93 Warren and Giles (n 48).

94 A review of SFDM for people with intellectual disabilities emphasized the importance of training and education to develop decision-making skills, with a focus on goal planning and self-regulation, as well as communication aids. S Werner, ‘Individuals with Learning Disabilities: A Review of Literature on Decision-Making since the Convention on the Rights of Persons with Disabilities (CRPD)’ Public Health Reviews 34 (2012) <https://publichealthreviews.biomedcentral.com/articles/10.1007/BF03391682> accessed 22 May 2025. Decision-making difficulties in the context of mental ill health has been linked emotional distress, and people with lived experience have highlighted the value of supporters affirming their capacity and instilling hope of recovery. (Amnesty International, ‘Decision-making Capacity in Mental Health: Exploratory Research into the Views of People with Personal Experience’ (Amnesty International Ireland 2009) <https://www.amnesty.ie/wp-content/uploads/2016/04/AI-Capacity-Research-Report.pdf> accessed 13 May 2025.) Advance decisions have been identified as a way of supporting decision-making for people with bipolar disorder. (L Stephenson and others, ‘Advance Decisions in Bipolar: A Systematic Review’ (2020) 11 Frontiers in Psychiatry <https://www.frontiersin.org/journals/psychiatry/articles/10.3389/fpsyt.2020.538107/full> accessed 15 May 2025.) See also: P Webb, ‘Key Components of Supporting and Assessing Decision Making Ability’ 72 (International Journal of Law and Psychiatry 2020) <https://www.sciencedirect.com/science/article/pii/S0160252720300728> accessed 25 May 2025.

95 SA Cooper and others, ‘Prevalence of and Associations with Mental Ill-health in Adults with Intellectual Disabilities’ (2007) 190 British Journal of Psychiatry 27.

96 D Enache and others, ‘Depression in Dementia: Epidemiology, Mechanisms, and Treatment’ (2011) 24 Current Opinion in Psychiatry 461; CQ Huang and others, ‘Cognitive Function and Risk for Depression in Old Age: A Meta-analysis of Published Literature’ (2011) 23 International psychogeriatrics 516.

97 D Mandell, ‘Dying before their Time: Addressing Premature Mortality among Autistic People’ (2018) 22 Autism 234.

98 Department for Constitutional Affairs (n 46) s 3.16.

99 Bigby and others (n 86).

100 K Samsi and J Manthorpe, ‘Everyday Decision-making in Dementia: Findings from a Longitudinal Interview Study of People with Dementia and Family Carers’ (2013) 25 International Psychogeriatrics 949.

101 L Knox, JM Douglas and C Bigby, ‘“I won’t be around forever”: Understanding the Decision-making Experiences of Adults with Severe TBI and their Parents’ (2016) 26 Neuropsychological Rehabilitation 236; L Knox, JM Douglas and C Bigby, ‘Becoming a Decision-making Supporter for Someone with Acquired Cognitive Disability Following Traumatic Brain Injury’ (2016) 3 Research and Practice in Intellectual and Developmental Disabilities 12; L Knox, JM Douglas and C Bigby, ‘“I’ve never been a yes person”: Decision-making Participation and Self-conceptualization after Severe Traumatic Brain Injury’ (2017) 39 Disability and Rehabilitation 2250.

102 L Luke and others, ‘Decision-making Difficulties Experienced by Adults with Autism Spectrum Conditions’ (2011) 16 Autism 612; E van der Plas, D Mason and F Happé, ‘Decision-making in Autism: A Narrative Review’ (2023) 27 Autism 1532.

103 Pathare and Shields (n 42); D Jeste and others, ‘Supported Decision Making in Serious Mental Illness’ (2018) 81 Psychiatry 28; L Brophy and others, ‘Community Treatment Orders and Supported Decision-making’ (2019) 10 Frontiers in Psychiatry 414; Kokanović and others (n 20); A Tinland and others, ‘Psychiatric Advanced Directives for People with Schizophrenia, Bipolar 1 Disorders, or Schizoaffective Disorders: Study Protocol for a Randomised Control Trial—DAiP Study’ (2019) 19 BMC Psychiatry <https://bmcpsychiatry.biomedcentral.com/articles/10.1186/s12888-019-2416-9> accessed 15 May 2025; Penzenstadler, Molodynski and Khazaal (n 20).

104 A document published by the British Psychological Society did include some SFDM advice tailored for the context of mental health conditions. C Herbert and others, *What makes a Good Assessment of Mental Capacity?* (The British Psychological Society 2019) 39 <https://ex plore.bps.org.uk/content/report-guideline/bpsrep.2019.rep127> accessed 14 May 2025.

105 This guide was recently updated. J Stavert, *Supported Decision-Making: Good Practice Guide* (Mental Welfare Commission for Scotland 2024) <https://www.mwcscot.org.uk/sites/default/files/2024-10/Supported%20Decision%20Making%202024.pdf> accessed 14 May 2025.

106 Department of Health, ‘Mental Health and Wellbeing Act 2022 Handbook’ (Victoria, Australia, Department of Health 2023) 21, 77–80. See also, L Brophy and others, ‘Guidelines for Supported Decision-making in Mental Health Services’ (RMIT University, Monash University, The University of Melbourne, 2018).

107 Royal Australian and New Zealand College of Psychiatrists, *Enabling Supported Decision-making* (RANZCP Victorian Branch Position Paper 2018).

108 Mental Health Foundation of New Zealand, *Embedding Supported Decision-making Across Aotearoa’s Mental Health System* (Mental Health Foundation of New Zealand 2023) <https://mentalhealth.org.nz/resources/resource/supported-decision-making> accessed 14 May 2025.

109 NHS England, ‘Acute Inpatient Mental Health Care for Adults and Older Adults’ (2023) <https://www.england.nhs.uk/long-read/acute-inpatient-mental-health-care-for-adults-and-older-adults/#summary> accessed 15 May 2025. The term ‘mental health’ here includes dementia, learning disability, autism, and drug or alcohol problems.

110 Kokanović and others (n 20). See also, JP Robertson and C Collinson, ‘Positive Risk Taking: Whose Risk Is It? An Exploration in Community Outreach Teams in Adult Mental Health and Learning Disability Services’ (2011) 13 Health, Risk and Society 147.

111 See Jeste (n 103) for a US perspective.

112 Department for Constitutional Affairs (n 46).

113 National Institute for Health and Care Excellence (n 11).

114 Warren and Giles (n 48).

115 Harding and Taşcıoğlu, ‘Everyday Decisions Project Report’ (n 3); Harding and Taşcıoğlu, ‘Supported Decision-Making from Theory to Practice’ (n 3).

116 Harding and Taşcıoğlu, ‘Everyday Decisions Project Report’ (n 3).

117 ibid.

118 Department for Constitutional Affairs (n 46).

119 Harding and Taşcıoğlu, ‘Supported Decision-Making from Theory to Practice’ (n 3) 12.

120 J Badger and R Parnell, ‘A Long Road To Travel: The Impact of the Mental Capacity Act on Adults with Complex Needs in Residential Settings’ (Scope, 2009) <https://www.mentalhealthlaw.co.uk/media/DH_Scope_Long_road_to_travel_Jan_2009.pdf> accessed 13 May 2025.

121 Harding and Taşcıoğlu, ‘Everyday Decisions Project Report’ (n 3) 27–29.

122 National Institute for Health and Care Excellence (n 11). We note, however, that approximately half of the identified resources were published prior to the NICE guidance.

123 Koldeweij and others (n 75) 1.

124 ibid 12.

125 Harding and Taşcıoğlu, ‘Everyday Decisions Project Report’ (n 3).

126 ibid 6.

127 Bigby and Douglas (n 37).

128 The Australian Royal Commission report states that, ‘The two most cited evidence informed frameworks are the La Trobe Support for Decision-making Practice Framework and the one by the Australian Cognitive Decline Partnership Centre, both of which were endorsed by study participants as good practice.’ Bigby and others (n 1) 8.

129 Bigby and Douglas (n 37) 14–15.

130 ibid 11–14.

131 ibid 16.

132 ibid 3.

133 Bigby and others (n 1).

134 Bigby and Douglas (n 37) 16.

135 Department of Health, *Providing Independent Advocacy under the Care Act* (The Stationary Office 2014) 117.

136 C Herbert and others, ‘Capacity to Consent to Sexual Relations’ (British Psychological Society, 2025) 38 <https://explore.bps.org.uk/content/report-guideline/bpsrep.2025.rep126a> accessed 14 May 2025.

137 Treating a relevant condition is found in the MCA Code, along with implementing a ‘programme to improve a person’s capacity to make particular decisions’, with the example of ‘helping a person with learning disabilities learn new skills’. Department for Constitutional Affairs (n 46) s 2.7. Education programmes are included in the NICE guidance. National Institute for Health and Care Excellence (n 11) s 1.2.9.

138 Harding and Taşcıoğlu, ‘Everyday Decisions Project Report’ (n 3) 4.

139 Warren and Giles (n 48) 7. The previous version of this guidance also referred to the CRPD. Fulton, Woodley and Sanderson (n 48).

140 British Medical Association (n 70). Guidance published by professional bodies such as the British Medical Association occupies an uncertain position in terms of its legal significance. Metcalf and colleagues observe that according to some commentators, such guidance carries less authority than documents produced by the government, NHS, or professional regulators, nonetheless, judges have sometimes accorded significant weight to BMA guidance. Metcalf, Pitkeathly and Herring (n 6).

141 British Medical Association (n 70) 12.

142 The Department of Health guidance notes that the MCA is ‘in some respects in tension with the empowering approach of Article 12’ (n 135) 113.

143 Committee on the Rights of Persons with Disabilities, ‘Concluding observations on the initial report of the United Kingdom of Great Britain and Northern Ireland’ (CRPD/C/GBR/CO/1, United Nations 2017) para 30.

144 MCA s 4.

145 Department of Health (n 135) 113.

146 British Medical Association (n 70) 12.

147 ibid.

148 ibid. The Court of Protection deals with cases involving the MCA.

149 Bartlett (n 16) 5. Bartlett also notes that, ‘at least some of the breaks between the current [MCA] system and an Article 12 compliant system may be less radical than the academic literature would suggest. Certainly, good practices that give voice to people with disabilities in these existing supportive decision-making arrangements, both in the “best interests” determination and in the capacity determination, should be identified and built upon’. Bartlett (n 16) 4.

150 BA Clough, ‘New Legal Landscapes: (Re)constructing the Boundaries of Mental Capacity Law’ (2018) 26 Medical Law Review 246, 272, 248. See Bartlett for consideration of an alternative way forward, in which ‘capacity is removed as a gateway concept’ in England and Wales (n 16, 5).

151 Harding and Taşcıoğlu, ‘Supported Decision-Making from Theory to Practice’ (n 3) 14.

152 Bigby and others (n 1) 63.

153 ibid.

154 ibid.

155 British Medical Association (n 70) 5.

156 ibid 12.

157 Bigby and others (n 1) 20–24.

158 British Association of Social Workers, ‘Capabilities Statement for Social Workers Working with Adults with Learning Disability’ (British Association of Social Workers, 2019) 16 <https://basw.co.uk/sites/default/files/resources/181064%20Learning%20Disability%20Capability%20Statement%2019.06.19.pdf> accessed May 13 2025.

